# Evaluating Causal and Noncausal Text Messages to Promote Physical Activity in Adults: Randomized Pilot Study

**DOI:** 10.2196/80090

**Published:** 2025-11-24

**Authors:** Elena Korshakova, Samantha Kleinberg

**Affiliations:** 1 Stevens Institute of Technology Hoboken, NJ United States

**Keywords:** causal information, text messaging, mHealth, physical activity, health communication

## Abstract

**Background:**

Physical inactivity increases the risk of chronic disease and reduces life expectancy, yet adherence to physical activity (PA) guidelines remains low. SMS text messages are promising for promoting PA, but it is not clear what type of messaging is most effective. Messages with causal information, which explain why a recommendation is being made, may be more persuasive than messages containing only recommendations.

**Objective:**

This study aims to compare the effectiveness of causal versus noncausal SMS text messages for promoting PA in US adults.

**Methods:**

In this pilot study, we randomized US adults (n=28 in the analytic sample) aged 18-64 years to receive causal or noncausal SMS text messages roughly every other day for 2 weeks, following a 1-week baseline. PA was measured using Empatica wristbands during intervention and baseline periods, and the International Physical Activity Questionnaire – Short Form (IPAQ-SF) at baseline, postintervention, and 4 weeks later. The primary outcome was the change in mean metabolic equivalent of tasks (METs) per minute from baseline to intervention. The secondary outcomes were (1) PA differences on intervention and nonintervention days (mean METs/min), (2) changes in self-reported METs per week between surveyed periods, and (3) participant satisfaction. We used a linear mixed model to analyze our primary outcome, the Mann-Whitney *U* test and the chi-square test of independence to analyze quantitative secondary outcomes, and qualitative coding to analyze survey data.

**Results:**

The causal message group had a greater increase in mean METs per minute from baseline to intervention compared to the noncausal group with a moderate effect size (*P*=.01; Cohen d=0.54). In the causal group, PA was significantly higher on SMS text message days (mean 2.46, SD 0.12 METs/min) compared to nonmessage days (mean 2.25, SD 0.15 METs/min; *P*=.02), while there was no difference in the noncausal group (*P*=.54). No significant between-group difference was found in self-reported PA or satisfaction.

**Conclusions:**

Causal information that links suggested PA to health outcomes can increase the effectiveness of SMS text messages promoting PA, indicating the value of incorporating causal information into intervention design. Our results provide further basis for just-in-time interventions, as activity was higher on message days. Further work is needed to better personalize message content and timing to maintain participant engagement.

## Introduction

Physical inactivity is associated with over 35 chronic medical conditions [1] and contributes to 8.3% of all-cause mortality in US adults, resulting in an estimated 320,000 annual deaths [2]. Despite widespread public health campaigns promoting regular physical activity (PA), adherence remains low. Sedentary behavior has increased among all age groups in the United States [3,4], a trend further exacerbated during the COVID-19 pandemic [5]. Thus, there is a need to address the increase in sedentary behavior [5,6].

To address the need to increase PA, many studies have explored mobile health interventions, particularly using SMS text messages. SMS text messages are cost-effective and can reach most of the US population, as 97% of US adults own a mobile phone capable of receiving SMS text messages [7]. Previous interventions have tested a variety of content types, such as motivating content about the benefits of PA [8] or providing reminders [9], with delivery varying in timing such as targeting morning due to higher motivation levels earlier in the day [10,11] or randomizing timing since responses may depend on individual timing patterns [12]. However, the effectiveness of SMS text message interventions in promoting PA has varied: 59% of studies reported no significant effects, and the remaining 41% demonstrated small to medium increases in PA [13]. The content of SMS text messages varied across studies, though the majority used motivational messages to encourage PA (76% of studies) or provided feedback (22% of studies) [13]. Given the mixed results of prior studies, there is a need to better understand what message characteristics have the largest influence on effectiveness.

One feature that has not been explored is the use of causal information, which explicitly links recommendations (stand up every 30 minutes) to outcomes (standing every 30 minutes can prevent chronic pain). Causal information could help individuals understand why a behavior matters. Recent research found that presenting causal information linking behavior to outcomes in a simple format, such as a 2-node causal diagram, can improve decision-making [14]. However, most experiments in this area have been conducted online using hypothetical decision-making scenarios, so it is unknown whether participants would replicate those choices in real-world situations. Relying on hypothetical scenarios can introduce an intention-behavior gap because intentions to take actions are not always translated into actual behavior [15]. Further, prior work has compared causal information to no information but has not compared causal versus noncausal messages.

To address this gap, we conducted a randomized pilot study to compare causal versus noncausal SMS text messages for increasing PA in real-world settings. Our primary outcome was the change in mean metabolic equivalent of tasks (METs) per minute from baseline to intervention. Secondary outcomes were (1) PA differences on intervention and nonintervention days (mean METs/min between message delivery and midnight), (2) changes in self-reported METs per week between surveyed periods, and (3) participant satisfaction.

## Methods

### Study Design

**Figure 1:**
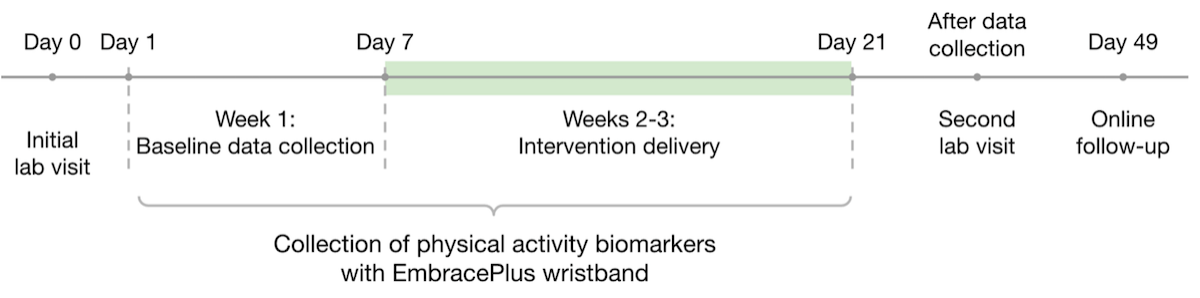
Data collection timeline for each participant.

We conducted a block-randomized pilot study comparing causal versus noncausal SMS text messages for promoting PA in real-world settings. Participants were assigned in blocks of 4 to either causal or noncausal SMS text messages, with participants being evenly allocated to each group. Data collection for each participant took place over 3 weeks: the first week being a baseline to determine usual PA, and the second 2 weeks being the intervention period. During the intervention weeks, participants received SMS text messages on a set schedule at a set time roughly every other day. PA was measured using objective (wristband tracking METs) and subjective (survey) instruments. Participants received a follow-up survey 4 weeks after completing their active study participation. Data collection occurred from November 2024 to July 2025. The overall study timeline is shown in Figure 1.

### Participants

We recruited individuals aged 18-64 years through community flyers (in New York City, Hoboken, and Jersey City) and a Craigslist posting. Eligibility criteria were no restrictions preventing participation in PA. We screened participants using an online form where participants completed the Physical Activity Readiness Questionnaire (PAR-Q+), a validated tool designed to identify individuals who may require medical clearance before starting an exercise program [16]. The questionnaire includes 7 yes/no questions about the participant’s health. If a participant answered “yes” to any of the questions, they were considered at risk and excluded from the study. Other exclusion criteria were inability to communicate in English, pregnancy or breastfeeding, plans to travel for an extended period during the study, or current participation in another PA intervention. Participants received a US $200 Amazon gift card after completing data collection and returning the devices, and a US $50 gift card after completing the follow-up survey.

### Interventions

Participants received either causal or noncausal SMS text messages roughly every other day at 8 AM for 2 weeks. We selected an every other day schedule to maintain engagement without causing message fatigue, and selected the 2-week duration based on prior work and the pilot nature of the study [13]. Message timing was selected to increase the likelihood that participants would see the messages early in the day and have sufficient time to include suggested activities into their daily routine. The message content was created based on PA guidelines developed by government agencies such as the US Centers for Disease Control and Prevention. Causal and noncausal texts were created in pairs such that each provided the same recommendation, with the difference being the causal messages included causal claims that linked recommendations to outcomes (eg, reducing risk of type 2 diabetes). Table 1 shows the full set of messages, delivery schedule, and source used to develop each message. Messages were automatically delivered according to our predetermined schedule. We used Textmagic to send and track messages, as it is a service that enables automated scheduling and delivery of texts, with confirmation of delivery. Participants were not required to reply to the messages, but during the consent meeting they were instructed to read them.

**Table 1:** Text message schedule and content by study day for each group, with sources used to develop each message.

Day	Causal group	Noncausal group
1 [17,18]	Engaging in moderate-intensity aerobic activities, like brisk walking, for at least 150 minutes every week helps reduce the risk of developing type 2 diabetes in adults. Let's get moving!	It is recommended that adults engage in moderate-intensity aerobic activities, like brisk walking, for at least 150 minutes every week. Let's get moving!
2 [19]	Taking a break from sitting every 30 minutes during your workday can help prevent chronic pain.	Take a break from sitting every 30 minutes during your workday.
4 [20]	Replacing sedentary time with physical activity of any intensity reduces the risk of developing chronic conditions such as arthritis.	It is recommended to replace sedentary time with physical activity of any intensity.
6 [18]	Incorporating moderate-intensity activities into your daily routine, such as brisk walking, biking on level ground or with minimal inclines, or pushing a lawn mower reduces the symptoms of depression and anxiety.	It is recommended to incorporate moderate-intensity activities into your daily routine, such as brisk walking, biking on level ground or with minimal inclines, or pushing a lawn mower.
8 [21]	Moving more and sitting less increases life expectancy. Stay active!	Health guidelines suggest moving more and sitting less. Stay active!
10 [17]	Adding physical activity to your workday by walking during your lunch break and taking the stairs when possible, boosts mental well-being by reducing stress and improving mood.	Add physical activity to your workday by walking during your lunch break and taking the stairs when possible.
12 [17]	By incorporating household chores like sweeping, vacuuming, and doing laundry into your daily routine, you can reduce the risk of heart disease.	Incorporate household chores like sweeping, vacuuming, and doing laundry into your daily routine.
14 [17]	Regular stair climbing improves heart health and helps lower blood pressure.	Take the stairs whenever you can.

### Procedures

Interested participants completed 2 online screening questionnaires: a demographic form (to assess eligibility based on age) and the PAR-Q+ (to assess eligibility for a PA intervention). Eligible participants were contacted via email and scheduled for an in-person consent meeting. During the meeting, participants were informed about the study procedures, given an opportunity to ask questions, and provided informed consent to participate. After consenting to the study, participants received both verbal and printed instructions on how to use the devices (smartphone and wristband), completed the International Physical Activity Questionnaire – Short Form (IPAQ-SF) to report baseline PA levels, provided a phone number for receiving SMS text messages, and selected a start date. Many prior studies assessed PA using MET scores from questionnaires or wearable devices [22,23]; thus, we used both measures in this study. Participants were encouraged to contact the investigators with any questions or technical difficulties with devices. After consent, participants were assigned to the causal or noncausal group.

For 3 weeks (1-week baseline and 2-week intervention), participants wore an activity tracking wristband on their nondominant wrist. Initially, the Empatica E4 wristband was used for measuring PA, but the device was discontinued on February 15, 2025. All participants enrolled after that date used the Empatica EmbracePlus wristband for data collection. Both wristbands have accelerometers and additionally capture temperature, electrodermal activity, and heart rate. In addition to the raw data, the EmbracePlus output includes biomarkers such as METs. To collect data from the wristband, participants used a provided Google Pixel 8A smartphone with the Empatica Care Lab app (for the EmbracePlus) or the E4 Realtime App (for the E4). While the wristband records activity, participants were also asked to press a button on the wristband each time they engaged in purposeful PA (eg, walking for exercise rather than for errands).

After completing the 3-week data collection period, participants returned to the lab. During a 30-minute visit, they returned the devices, completed the IPAQ-SF again, and were interviewed about their satisfaction with the intervention through a series of multiple-choice and open-ended questions.

Finally, 4 weeks after completing data collection, participants completed an online follow-up questionnaire to assess the long-term effects of the intervention using the IPAQ-SF.

### Outcomes

#### Objective PA Assessment

The primary study outcome and one secondary outcome focused on PA measured using METs. We selected METs because it is a widely accepted measure of PA intensity and captures energy expenditure across a range of activities, including both formal exercise and everyday tasks like household chores and stair climbing, as suggested in our SMS text messages. The wristband continuously recorded METs and sent data to the app via Bluetooth. When connected to Wi-Fi, the app uploaded data to the cloud, which enabled us to track compliance. Participants wore the wristband during all waking hours and were instructed to charge it overnight. Participants did not receive any feedback from the wristband, which only displayed time of day (EmbracePlus) or had no display (E4), and participants did not see any of the data being collected.

#### Self-Reported Measures

Our last two secondary outcomes focused on self-reported measures. First, we collected self-reported PA using the IPAQ-SF at 3 time points: before baseline data collection, after completion of the intervention period, and 4 weeks postcompletion. This questionnaire assesses PA categorically (low, medium, and high) and through a continuous score (calculated as a MET level multiplied by the number of minutes of activity and events per week). Due to the potential bias of self-reporting [24], this measure was used as a secondary outcome. Additionally, we surveyed participants about their satisfaction with the intervention using a combination of multiple-choice and open-ended questions. The full survey is in Multimedia Appendix 1.

### Sample Size

Due to the preliminary nature of the study, we did not use an a priori power calculation. Based on sample sizes used in prior work to evaluate similar types of interventions [13], we set a sample size of 30 to assess feasibility and estimate effect sizes for future trials.

### Randomization

We used block randomization with a block size of 4 to ensure balanced group sizes and distribution of assignment over time. The sequence was generated by the first author (EK).

### Blinding

Our primary outcome used METs recorded automatically by the wristband. Participants did not have access to the recorded data, so assessment of outcomes was not influenced by expectations of participants or the investigators. Due to the nature of the intervention, it is not possible to conceal message content from participants or investigators, and data analysis was not blinded.

### Analysis

To evaluate differences in demographics between groups, we used a chi-square test of independence, which is suitable for small sample sizes. We used a Mann-Whitney *U* test to compare baseline PA between groups in mean METs per minute, as the MET data were not normally distributed.

The primary outcome was defined as the change in mean METs/min between baseline and the 2-week intervention period. We used a linear mixed model (LMM) with fixed effects of group (causal and noncausal), age, and gender. Our target variable was calculated as the difference between participant-level mean METs/min during the intervention and baseline periods. We used METs/min due to varying durations of recording between and within participants (eg, differing number of waking hours) and incomplete hours (eg, starting recording at 8:56 AM means only 4 minutes of data would be available for that hour). We used random intercepts for participants to account for individual differences in baseline activity, and a random slope for week to allow variation in week-to-week change trajectories across individuals. Random effects were grouped by participant ID. The model was fit using restricted maximum likelihood estimation. We selected LMM as it is appropriate for analyzing repeated measurements taken over time under different conditions and was used in prior work [22]. To quantify the effect size of the between-group difference, we calculated the Cohen *d* score.

Our first secondary outcome focused on differences between message and nonmessage days between groups. Since messages were all delivered at 8 AM local time, we calculated METs/min between 8 AM and 12 AM for each day of the intervention period for each person, and took the mean of message and nonmessage days. We used the Mann-Whitney *U* test to compare mean METs/min on message and nonmessage days.

Self-reported PA was calculated as mean METs per week based on participants’ responses to the IPAQ-SF. This measure reflects the frequency and duration of different intensities of activity reported over the past 7 days. The Mann-Whitney *U* test was used to compare the reported PA in mean METs per week between each survey.

Lastly, we analyzed participants’ responses to the satisfaction questionnaire using the mean for each response option. We used a chi-square test of independence to compare whether the differences in responses were statistically significant. For qualitative data, we conducted a bottom-up (inductive) thematic analysis. The first author read all responses first, then manually assigned descriptive codes to specific comments (eg, “Something more personalized like facts about you and your physical activity” was coded as “lack of data integration”). Similar codes were then grouped into broader themes (eg, the theme “lack of personalization”), and we calculated the frequency of mentions for each subtheme by group.

### Ethical Considerations

The study was approved by the institutional review board (IRB) of Stevens Institute of Technology (IRB protocol #2024-052). All participants provided informed consent before data collection. We maintain privacy of all participants and do not include any identifying information. Participants received a US $200 Amazon gift card after completing data collection and returning the devices, and a US $50 gift card after completing the follow-up survey.

## Results

### Participants

**Figure 2:**
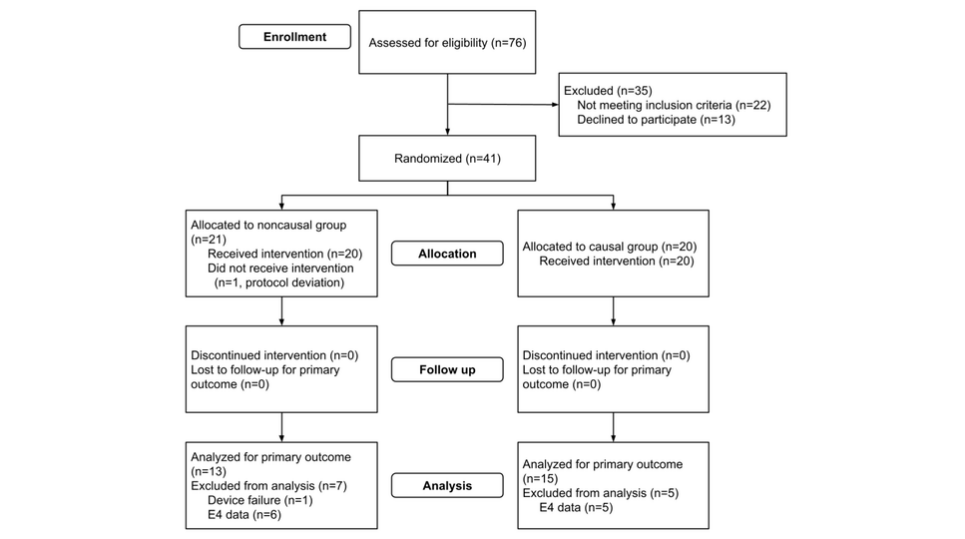
CONSORT (Consolidated Standards of Reporting Trials) diagram. Exclusions shown are for participants completely excluded from analysis. Only participants who used the EmbracePlus were included in the analysis. Partial exclusions are described in the text.

As shown in Figure 2, a total of 76 individuals completed the screening questionnaire and provided their email addresses for follow-up. Of these, 41 were consented and completed data collection. After examining the data collected with the E4, we observed significant missing data due to the devices frequently disconnecting from the app. Since each participant used a single device for all data collection (baseline and intervention), we thus excluded all participants who used the E4 from analysis. This decision was made prior to any data analysis and led to continuing enrollment until meeting our predetermined target of 30 participants with the EmbracePlus. Thus, 13 participants were excluded from all analyses (n=1 due to protocol deviation, n=1 due to device failure leading to over 5 days of missing data, and n=11 due to data collection using the E4), resulting in a final sample of 28 participants.

Participants’ demographics and baseline activity by group are shown in Table 2, and there were no significant differences between groups. One participant did not complete the follow-up survey and was excluded from analysis of that data (and thus received partial payment for the study, US $200). A total of 588 days of data were collected and included in the analysis. Five days were excluded: 4 participants each had 1 missing day, with 1 day being during the baseline period and 3 days during the intervention period (1 on a day when a message was delivered, and the other 2 on a day without a message). Additionally, 1 participant reported completing an unplanned 32-mile walk during the baseline week. Since the baseline week was intended to represent usual behavior, the 32-mile walk was considered abnormal and that day was excluded, with means then calculated based on the present days.

**Table 2:** Participant demographics and baseline physical activity. Values are presented as n or mean (SD). *P*<.05 was considered statistically significant.

Variables			Causal (n=15)		Noncausal (n=13)		*P* value
**Gender, n**							.76
	Male	10		7			
	Female	5		6			
Age in years, mean (SD)			35 (10.49)		35.18 (10.67)		.89
**Race, n**							.31
	White	4		5			
	Asian	7		2			
	Black or African American	2		2			
	Hispanic, Latino/a, or Spanish Origin	2		2			
	Asian and Black or African American	0		1			
	Black or African American and Hispanic, Latino/a, or Spanish Origin	0		1			
**Education, n**							.38
	High school	1		3			
	Undergraduate degree	6		3			
	Graduate degree	8		7			
Baseline physical activity (METs^a^ per minute), mean (SD)			2.31 (0.47)		2.38 (0.51)		.61

^a^METs: metabolic equivalent of tasks.

### Primary Outcome: Difference in PA Between the Baseline and Intervention Periods

Our primary outcome was the difference in mean METs/min between the baseline and intervention period. As shown in Table 3, there was a significant effect of group, indicating that the increase in the causal group was significantly different. The effect size was moderate (Cohen *d*=0.54), indicating a meaningful difference between the causal and noncausal groups. Additionally, there was a significant effect of gender, with women being more likely to increase PA from baseline to the intervention period compared to men. Table 4 reports the mean METs during the baseline and intervention periods. An increase was observed in the causal group and a decrease in the noncausal group; however, neither difference was statistically significant.

**Table 3:** Results for linear mixed model predicting change in mean metabolic equivalent of tasks (METs) per minute from baseline to intervention period. Coefficients show the change in METs for a one-unit increase in each predictor, with positive coefficients meaning an increase in METs. *P* values <.05 were considered statistically significant. N=28 for this analysis.

Predictor	β (95% CI)	SE	*z* score	*P* value
Intercept	–0.12 (–0.18 to –0.06)	0.03	–4.05	<.001
Group (causal)	0.13 (0.04 to 0.22)	0.05	2.74	.01
Gender (woman)	0.13 (0.01 to 0.26)	0.06	2.11	.04
Age	0.00 (–0.01 to 0.01)	0.00	0.34	.73

**Table 4:** Mean (SD) metabolic equivalent of tasks per minute during baseline and intervention periods (compared using the Mann-Whitney U test). N=28 for this analysis.

	Baseline	Intervention	*P* value
Causal	2.31 (0.47)	2.40 (0.47)	.51
Noncausal	2.38 (0.51)	2.37 (0.46)	.99

### Comparing PA on Days With and Without Message Delivery by Group

Our primary outcome was overall activity change. We next examined differences in PA between days with and without SMS text message delivery. Participants in the causal group were significantly more active on days when they received SMS text messages (mean 2.46, SD 0.12) compared to days when they did not (mean 2.25, SD 0.15; *U*=6; *P*=.02). The difference was not significant in the noncausal group (days with texts: mean 2.40, SD 0.26; days without texts: mean 2.33, SD 0.19; *U*=19; *P*=.57).

### Changes in Self-Reported PA

Based on self-reported data, baseline MET-minutes/week did not differ between participants in the causal group (mean 1661.82, SD 1026.94) and the noncausal group (mean 2316.75, SD 1562.08; *U*=48; *P*=.29; n=27). Both groups reported an increase in MET-minutes/week from baseline to intervention, though this increase was not significantly different between the causal (mean 1037.49, SD 1501.28) and noncausal groups (mean 369.45, SD 366.45; *U*=94; *P*=.15). At 4 weeks after the intervention, MET-minutes/week did not change significantly from baseline in either the causal (mean 372.12, SD 986.39; *U*=68; *P*=.36) or noncausal group (mean 83.21, SD 423.76; *U*=78; *P*=.42).

### Participant Satisfaction

We first analyzed the quantitative survey data. As shown in Table 5, while there were some differences, none were significant between the two groups. Most participants in both groups reported that they always read the messages and found them easy or very easy to understand. While more participants reported satisfaction with text frequency in the causal compared to the noncausal group, the difference was not significant. Similarly, 8 participants in the causal group reported that the messages helped them better achieve their fitness goals, while only 2 participants in the noncausal group reported the same.

Lastly, we examined participants’ answers to the open-ended questions using thematic analysis, as shown in Table 6. Across all participants (N=28), 15 were dissatisfied with the frequency of message delivery and expressed a preference for more messages. Six participants in the causal group indicated that references to specific health outcomes, such as diabetes and mental health, made the messages motivating.

The reminders of specific consequences like diabetes and chronic pain really stuck with me. Every message felt like a gentle but effective nudge toward being more active.

Participant 7

In the noncausal group, 4 participants mentioned that the messages were too generic and easy to ignore or forget.

The messages were too generic and failed to capture my attention.

Participant 12

Participants in both groups expressed positive views about the messages as helpful reminders. Three participants across both groups mentioned that they would prefer a friendlier, more conversational tone in the messages.

I think it's the copywriting that needs to be more conversational. It sounded too sterile and cold.

Participant 9

Participants in both groups mentioned a lack of personalization (n=22), expressing a preference for receiving messages specifically after periods of inactivity, when the reminder would be more relevant.

Messages could be more immediate when you don't move for a ling [sic] time.

Participant 4

**Table 5:** Participants’ responses to the satisfaction questionnaire by group.

Question		Causal (n=15), n	Noncausal (n=13), n	*P* value
**How often did you read the text messages?**				.99
	Always	13	12	
	Often	2	1	
	Sometimes	0	0	
	Rarely	0	0	
	Never	0	0	
**Did the frequency of the text messages meet your expectations?**				.24
	Yes	9	4	
	No	6	9	
**Did the text messages help you better achieve your fitness goals?**				.09
	Yes	8	2	
	No	7	11	
**Did the text messages help you feel more motivated to make healthy lifestyle changes?**				.28
	Yes	11	6	
	No	4	7	
**How easy was it to understand the information provided in the text messages?**				.83
	Very easy	12	9	
	Easy	3	4	
	Neither easy nor difficult	0	0	
	Difficult	0	0	
	Very difficult	0	0	
**Would you recommend this text message program to a friend?**				.27
	Yes	13	8	
	No	2	5	
**How likely are you to continue using the strategies or advice provided in the text messages after the study?**				.30
	Very likely	6	1	
	Likely	7	8	
	Neither likely nor unlikely	1	1	
	Unlikely	1	2	
	Very unlikely	0	1	
**Overall, how satisfied were you with the convenience and accessibility of receiving health information via text messages?**				.18
	Very satisfied	10	6	
	Satisfied	3	6	
	Neither satisfied nor dissatisfied	2	0	
	Dissatisfied	0	1	
	Very dissatisfied	0	0	

**Table 6:** Themes and subthemes identified from participants' answers to open-ended questions. Numbers in the last two columns represent the number of mentions by group.

Theme and subtheme		Causal (n=15)	Noncausal (n=13)
**Frequency**			
	Preference for more frequent messages	6	9
**Message content**			
	Motivating messages	6	0
	Generic content	0	4
	Reminders	5	4
	Tone of messages	2	1
	Educational content	2	0
	Already known information	0	2
**Lack of personalization**			
	Lack of data integration	6	4
	Inconvenient delivery timing	3	4
	Lack of personalized feedback	2	3
**Message features**			
	Not interactive	1	2
	Not tied to real-time behavior data	2	1
**Technology and usability**			
	App issues	1	0
	Technical or connectivity issues	1	0
**Recommendation logic**			
	Better for inactive users	2	3
	Not helpful for already active users	1	2
**Other**			
	No changes needed	3	2
	No feedback	2	1

## Discussion

### Principal Results

We found that causal messages were more effective than noncausal ones, leading to a statistically significant increase in PA with a moderate effect size. Analysis of the secondary outcomes showed that participants in the causal group were more active on days when they received messages than on days when they did not. Potentially due to the small sample size, there were no significant differences in self-reported PA increase between groups or time periods, and no significant differences in satisfaction were found.

### Comparison With Prior Work

We developed causal messages to provide a clear rationale for behavior change by linking PA to meaningful health outcomes, such as a reduced risk of chronic diseases like type 2 diabetes and improved mental health. This approach was effective for increasing PA, and our qualitative results suggest that the causal component may be especially motivating. Our findings advance prior work on SMS text messaging for increasing PA by identifying for the first time that causal messages may be more effective than statements that only recommend an activity. Our results align with previous research suggesting that individuals are more likely to engage in a behavior when they understand its relevance and how it impacts their overall well-being [25]. By explicitly connecting PA to potential health outcomes, causal messages may have increased perceived relevance and personal significance [26]. Similarly, Fogg’s Behavior Model emphasizes that increasing the perceived importance of an action can serve as a trigger for behavior change [27].

Participants in the causal group were significantly more active on days when they received messages, and dissatisfaction with message frequency was due to a desire for more messages rather than burnout. Our messaging schedule was designed to avoid message fatigue, but our results show that more frequent messages could be both acceptable and effective. Prior research suggests that individualized or adaptive message schedules are often more effective than fixed-frequency approaches [28]. Our results suggest that consistent exposure to causal messages may promote more sustainable engagement in PA. However, it is an open question whether these results would differ in a long-term intervention, as our study period was 2 weeks. Although more than half of the participants expressed a preference for more frequent messages, allowing people to customize message frequency according to their needs may increase the effectiveness of future interventions. Participants also mentioned a preference for receiving messages after prolonged periods of inactivity. This aligns with existing evidence that timely, context-sensitive prompts can positively influence behavior in real time [29]. Thus, it may be important to balance regular message delivery with personalized scheduling to optimize engagement and minimize fatigue in future PA interventions.

The increase in self-reported PA in our study was not significant from baseline to the intervention period and 4 weeks postintervention between groups, which may be due to the small sample size. Further, the self-reported values were inconsistent with the objective measures, where we found a significant increase in METs in the causal group and no increase in the noncausal group. This discrepancy is consistent with prior research showing that self-reported PA is often overestimated and may not accurately reflect actual behavior [30,31]. Our self-reported measures and METs measured with the EmbracePlus are not directly comparable, but the difference in trends highlights the importance of using objective measures when evaluating the effectiveness of behavioral interventions.

Our findings were supported by insights from the participant surveys. Participants reported that causal messages linking PA to health outcomes increased their motivation to be physically active. Additionally, we found that most participants wanted messages to be more personalized and timelier, based on their PA data. Since our work was a pilot, future work is needed to explore whether causal framing is still effective when combined with personalized PA data to deliver messages during periods of inactivity. Interventions could consider individuals’ calendars, targeting moments when they are available to take a break and engage in PA.

### Limitations

Our study had several limitations. The sample size for this pilot study was small and consisted of residents from New York and New Jersey, which may limit the generalizability of our findings to broader populations. The intervention period was only 2 weeks, and longer-term adherence and outcomes remain unknown. This is especially important given our finding that participants wanted more frequent messages. Since messages were on a set schedule, it is not known whether tailoring them to individual activity levels would have benefits beyond that of causal messaging, nor whether varying the schedule is important for long-term engagement. Future research is needed to explore the long-term effectiveness of causal messaging, test more personalized message delivery schedules, and examine the use of interventions based on real-time activity data. Finally, due to the nature of the intervention and pilot study, allocation could not be fully concealed. While our use of objective measures limits the potential for bias in outcome assessment, this is nevertheless a limitation.

### Conclusions

We found that incorporating causal information into an SMS text message intervention for promoting PA can increase their effectiveness. Specifically, participants who received messages that included causal information linking recommended PA to health outcomes showed a greater increase in PA from baseline compared to those who received the messages with the same PA recommendations without causal information, and participants in the causal group had more PA on days with messages than on days without. These findings have important implications for the design of future behavioral interventions. We recommend incorporating causal information that explicitly connects PA recommendations to relevant health outcomes, such as reduced risk of chronic diseases. Moreover, tailoring message delivery to participants' preferences and real-time context may support sustained engagement and increase the intervention’s effectiveness.

## Appendix

Multimedia Appendix 1 Survey questionnaire.

## Abbreviations

IPAQ-SF: International Physical Activity Questionnaire – Short Form
IRB: institutional review board
LMM: linear mixed model
MET: metabolic equivalent of task
PA: physical activity
PAR-Q+: Physical Activity Readiness Questionnaire

## Data Availability

The datasets generated or analyzed during this study are available from the corresponding author on reasonable request.
