# Supplementary material for: Evaluating Causal and Noncausal Text Messages to Promote Physical Activity in Adults: Randomized Pilot Study
Source: JMIR Form Res. 2025 Nov 24;9:e80090. doi: 10.2196/80090 (PMC12686858; doi:10.2196/80090)
Supplement: Multimedia Appendix 1 [file formative_v9i1e80090_app1.docx]

# Survey questionnaire

1. How often did you read the text messages?

- Always
- Often
- Sometimes
- Rarely
- Never

2. Did the frequency of the text messages meet your expectations?

- Yes
- No

2.1. If no, would you have preferred more or fewer messages? (Open-ended)

3. Did the text messages help you better achieve your fitness goals?

- Yes
- No

3.1. If yes, in what ways did they help? (Open-ended)

3.2. If not, could you explain why? (Open-ended)

4. Did the text messages help you feel more motivated to make healthy lifestyle changes?

- Yes
- No

4.1. If yes, how did they help? (Open-ended)

4.2. If not, could you explain why? (Open-ended)

5. How easy was it to understand the information provided in the text messages?

- Very easy
- Easy
- Neither easy nor difficult
- Difficult
- Very difficult

6. Would you recommend this text message program to a friend?

- Yes
- No

6.1. Why or why not? (Open-ended)

7. How likely are you to continue using the strategies or advice provided in the text messages after the study?

- Very likely
- Likely
- Neither likely nor unlikely
- Unlikely
- Very unlikely

8. Overall, how satisfied were you with the convenience and accessibility of receiving health information via text messages?

- Very satisfied
- Satisfied
- Neither satisfied nor dissatisfied
- Dissatisfied
- Very dissatisfied

9. If you could change one thing about the text messages, what would it be? (Open-ended)

10. Is there any other feedback you would like to share with us about participating in the study? (Open-ended)
